# Performance evaluation of a rapid whole-blood immunoassay for the detection of IgG antibodies against *Helicobacter pylori* in daily clinical practice

**DOI:** 10.1186/s12941-016-0161-1

**Published:** 2016-08-08

**Authors:** Dietmar Enko, Gabriele Halwachs-Baumann, Robert Stolba, Ortrun Rössler, Gernot Kriegshäuser

**Affiliations:** 1 Institute of Clinical Chemistry and Laboratory Medicine, General Hospital Steyr, Sierningerstraße 170, 4400 Steyr, Austria; 2 Institute of Pathology, General Hospital Steyr, Sierningerstraße 170, 4400 Steyr, Austria

**Keywords:** *Helicobacter pylori*, Immunoassay, Laboratory diagnosis

## Abstract

**Background:**

A growing number of rapid *Helicobacter pylori* antibody tests are commercially available now, however, some of these tests are often used without sufficient evaluation. The aim of this study was to evaluate the performance of a commercially available rapid whole-blood immunoassay (gabControl^®^*H. pylori*; gabmed GmbH, Köln, Germany), for the qualitative detection of IgG antibodies against *H. pylori* with the ^13^C-urea breath test (^13^C-UBT) serving as a reference method.

**Methods:**

A total of 108 consecutive outpatients, who were referred for ^13^C-UBT by general practitioners and specialists, were also tested for *H. pylori* infection by the gabControl^®^*H. pylori* immunoassay. The clinical performance of this rapid whole-blood test was evaluated by determining the sensitivity, specificity, positive predictive value (PPV), and negative predictive value (NPV) compared to the ^13^C-UBT. The agreement between the two tests was calculated using Cohen’s Kappa (κ) with 95 % confidence intervals (CI).

**Results:**

The agreement between the gabControl^®^*H. pylori* assay and the ^13^C-UBT was 0.62 [95 % confidence intervals (CIs) 0.47–0.76; P < 0.001]. With the ^13^C-UBT serving as the non-invasive gold standard method of *H. pylori* diagnosis, the gabControl^®^*H. pylori* assay demonstrated a sensitivity and specificity of 91.4 and 76.7 %, respectively, with a PPV of 65.3 % and a NPV of 94.9 %. Seventeen (15.7 %) individuals with a positive *H. pylori* anamnesis showed a negative ^13^C-UBT and were typed positive by the gabControl^®^*H. pylori* assay. Of these, 13 (76.5 %) and 3 individuals (17.6 %) had completed one and two eradication therapies, respectively.

**Conclusions:**

The gabControl^®^*H. pylori* immunoassay is a rapid and easy to use first line screening tool for *H. pylori* IgG antibody detection in daily clinical practice. However, this assay should not be used for confirmation of the successful *H. pylori* eradication after antibiotic treatment.

## Background

*Helicobacter pylori* infection is still a common condition worldwide. In North Europe and North America, about one-third of adults are infected, whereas in South-East Europe, South America, and in Asia, the *H. pylori* prevalence is reported to be higher than 50 % [1].

Since the *H. pylori* infection was recognized as a causative agent of chronic active gastritis and a risk factor for ulcer disease, gastric cancer and the mucosa-associated lymphoid tissue (MALT) lymphoma, numerous invasive and non-invasive methods for the accurate detection of this bacterium have been developed. Invasive techniques include biopsy-based histological methods, culture of the bacterium, the rapid urease test, and molecular tests (e.g. real-time PCR). Non-invasive methods encompass the ^13^C-urea breath test (^13^C-UBT), the stool antigen test, and the *H. pylori* antibody detection by serological tests [2–4].

The ^13^C-UBT is considered the non-invasive gold standard method of *H. pylori* diagnosis [5–7]. It is a simple and safe test, which is easily repeated and provides excellent accuracy for the initial diagnosis of *H. pylori* infection, as well as the confirmation of its eradication after treatment [7, 8]. In the presence of the *H. pylori* produced enzyme urease, the ingested labeled urea (^13^C-urea) is metabolized into labeled carbon dioxide (^13^CO_2_) and ammonia (NH_3_). The produced ^13^CO_2_ diffuses into the blood vessels and is eliminated via the lungs. The expired air is collected in order to measure the activity of labeled carbon so as to detect individuals with *H. pylori* infection [5, 9, 10].

Since individuals infected with *H. pylori* develop a local and systematic immune response [11, 12], specific *H. pylori* antibodies can be detected by rapid serological assays. These tests are easy to perform, inexpensive, and enable immediate patient testing for *H. pylori* antibodies in general practice surgeries [13]. A previous study, which evaluated a rapid whole-blood test, demonstrated, that there was no difference in diagnostic accuracy between capillary (fingerstick) and venous blood (venipuncture) collection [14]. A growing number of rapid *H. pylori* antibody tests are commercially available now, however, some of these tests are often used without sufficient evaluation.

The aim of this study was to evaluate the performance of a commercially available rapid whole blood immunoassay (gabControl^®^*H. pylori*; gabmed GmbH, Köln, Germany), for the qualitative detection of IgG antibodies against *H. pylori* with the ^13^C-UBT serving as a reference method.

## Methods

### Patients

In total, 108 patients, who were consecutively referred for ^13^C-UBT by general practitioners and specialists to our outpatient clinic, were also tested for *H. pylori* infection by the gabControl^®^*H. pylori* immunoassay (gabmed GmbH, Köln, Germany). The study period was from January to December 2015. The inclusion criteria were a minimum age of >15 years, an overnight fasting state and a non-smoking period >12 h before the ^13^C-UBT. Patients with antibiotic-based therapy at least 4 weeks before and/or proton pump inhibitor (PPI) therapy at least 2 weeks before the ^13^C-UBT were excluded from the study. An anamnesis was carried out about the history of *H. pylori* infections, completed eradication therapies, and intake of medication. Written informed consent was provided from all the patients. The ethical approval for this study was obtained from the Ethical Committee of Upper Austria, Linz, Austria. The study was carried out in accordance with the latest version of the Declaration of Helsinki.

### ^13^C-UBT

Isotope ratio mass spectrometry was employed using the IRIS^®^-^13^C-Infrared Isotope Analyzer System (Wagner Analysen Technik GmbH, Bremen, Germany). The ^13^C-UBT was performed according to the manufacturer’s instructions. Briefly: after a 12 h fasting period, breath samples were obtained before (baseline) and 30 min after the test drink intake (75 mg ^13^C-urea from the capsule dissolved in 200 mL fruit juice) early in the morning (8:00–10.00 a. m.). ^13^C/^12^C-isotope ratio differences between the value at 30 min and the baseline value were determined and expressed in delta over baseline (DOB, ‰). A sample was considered positive if the 30 min value was above a 4 ‰ cut-off level [15]. Eating, drinking and/or smoking were not allowed until the ^13^C-UBT was completed.

### GabControl^®^*H. pylori*

**Fig. 1:**
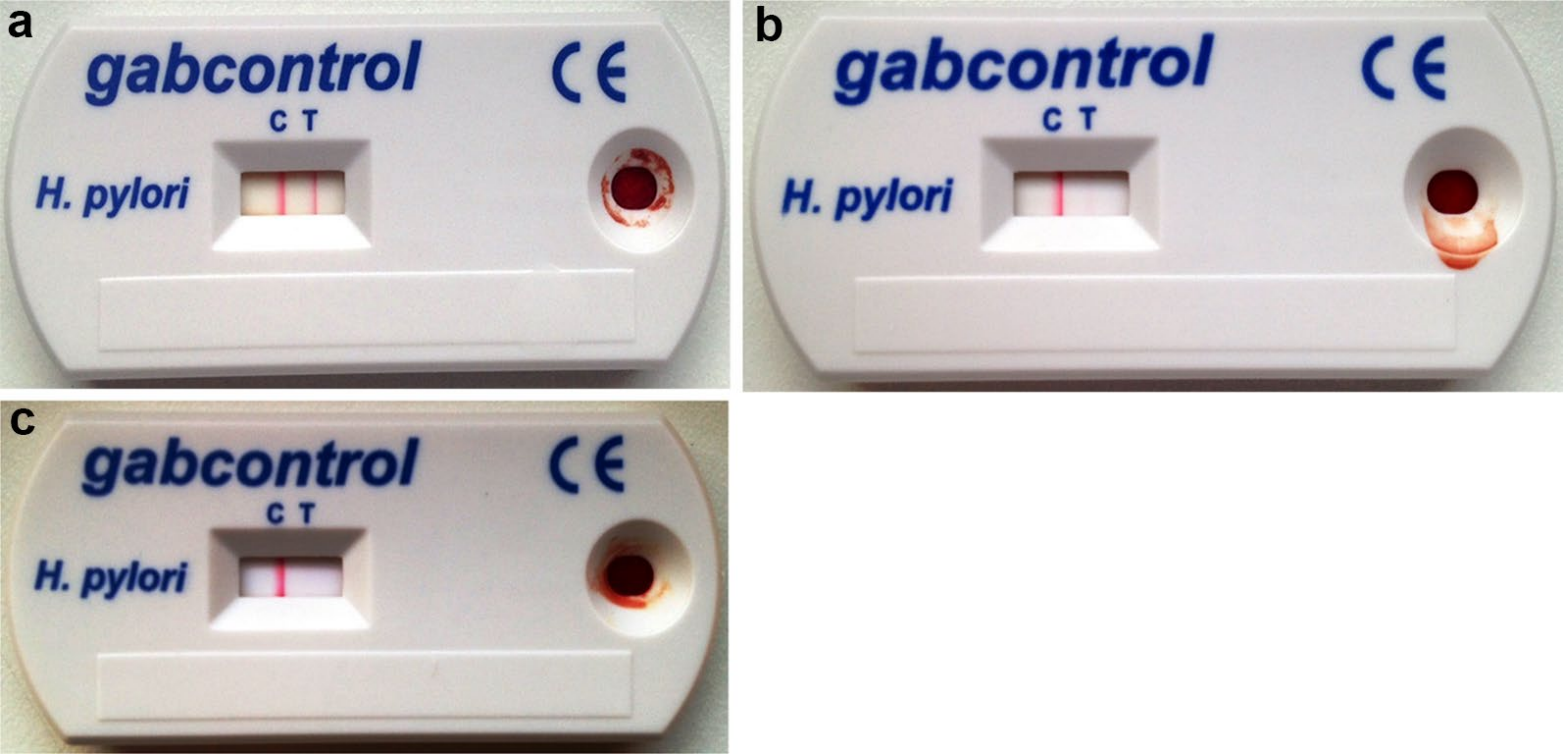
Typical reactivity patters obtained for the gabControl^®^ *H. pylori* immunoassay: **a** thirty-four (31.5 %) individuals showed an intense T line signal, **b** 15 (13.9 %) individuals were observed with faint to faintest T line signals, **c** 59 (54.6 %) patients demonstrated a C line signal only. *C* control line; *T* test line

This commercially available test is a qualitative membrane based immunoassay for the qualitative detection of *H. pylori* IgG antibodies in whole-blood, serum or plasma. The test was performed in accordance with the manufacturer’s instructions. Approximately 50 µL of fingerstick whole-blood was sampled in a glass capillary tube and transferred to the specimen well (S) on the test device (Fig. 1a–c). One drop (approximately 40 µL) of dilution buffer containing *H. pylori* antigen-coated particles was added and allowed to migrate along a lateral-flow membrane thereby interacting with anti-human IgG antibodies immobilized as parallel lines. The test results were read after 10 min: a red control (C) line signal together with a red test (T) line signal (intense or faint) indicated the presence of *H. pylori* IgG antibody (Fig. 1a, b); showing a single red C line signal only, the assay was interpreted as negative (Fig. 1c). Assay read-out was performed independently by two physicians (i.e. four eyes principle), which were both blinded to the respective ^13^C-UBT result.

### Statistical analysis

The agreement between the ^13^C-UBT and the gabControl^®^*H. pylori* assay was calculated using Cohen’s Kappa (κ) with 95 % confidence intervals (CIs) [16]. Sensitivity, specificity, positive predictive value (PPV) and negative predictive value (NPV) of the gabControl^®^*H. pylori* assay were calculated compared to the ^13^C-UBT. No adjustment for type I error was made. Therefore the concerning P values are only descriptive. Analyse-it^®^ software version 2.30 (Analyse-it Software, Ltd, Leeds, United Kingdom) was used for statistical analysis.

## Results

### Study population characteristics

Of 108 consecutively enrolled patients, 36 (33.3 %) were male and 72 (66.7 %) were female. The median age was 44.0 (range: 15–88) years. The main demographic and clinical characteristics of the study population are provided in Table 1. Fifty-eight (53.7 %) individuals had a positive history of previous *H. pylori* infections.

**Table 1:** Basic characteristics of the study population

	Study population
Patients	n = 108
*Gender*	
Male	n = 36 (33.3 %)
Female	n = 72 (66.7 %)
Median age (years)	44 (range: 15–88)
*History of previous H. pylori infections*	
Negative history	n = 50 (46.3 %)
Positive history	n = 58 (53.7 %)
Completed eradication therapies (one or more)	n = 54 (50 %)

### Performance of the gabControl^®^*H. pylori* assay

The gabControl^®^*H. pylori* assay was found positive and negative in 49/108 (45.4 %) and 59/108 (54.6 %) patients, respectively (Table 2). Of those patients with a positive result, fifteen (30.6 %) individuals showed faint red colored changes in the test line region (T) only (Fig. 1b).

**Table 2:** GabControl^®^ *H. pylori* versus ^13^C-UBT

	n = 108	
	GabControl^®^ *H. pylori* ^a^ +	GabControl^®^ *H. pylori* ^a^ −
^13^C-UBT +	32 (29.6 %)	3 (2.8 %)
^13^C-UBT −	17 (15.7 %)	56 (51.9 %)

^a^ The gabControl^®^ *H. pylori* immunoassay showed a sensitivity of 91.4 % and a specificity of 76.7 % with the ^13^C-UBT serving as a reference method

The agreement between the gabControl^®^*H. pylori* assay and the ^13^C-UBT was 0.62 (95 % CI 0.47–0.76; P < 0.001). With the ^13^C-UBT serving as the non-invasive gold standard method of *H. pylori* diagnosis [5–7], the gabControl^®^*H. pylori* assay demonstrated a sensitivity and specificity of 91.4 and 76.7 %, respectively, with a PPV of 65.3 % and a NPV of 94.9 % (Table 2).

Thirty-five (32.4 %) individuals had a positive ^13^C-UBT. Twenty-seven (77.1 %) of these patients had a positive *H. pylori* anamnesis, whereas in 8 patients (22.9 %) *H. pylori* infection was detected for the first time. Seventeen (15.7 %) individuals with a positive *H. pylori* anamnesis showed a negative ^13^C-UBT and were typed positive by the gabControl^®^*H. pylori* assay. Of these, 13 (76.5 %) and 3 individuals (17.6 %) had completed one and two eradication therapies, respectively. One patient (5.9 %), however, did not undergo any kind of antibiotic treatment.

## Discussion

This study aimed to compare the performance of the gabControl^®^*H. pylori* rapid immunoassay with the ^13^C-UBT in 108 patients, who were referred by general practitioners and specialists to our outpatient clinic. As the ^13^C-UBT was considered the non-invasive gold standard method of *H. pylori* diagnosis [5–7], the gabControl^®^*H. pylori* assay demonstrated a sensitivity and specificity of 91.4 and 76.7 %, respectively, with a PPV of 65.3 % and a NPV of 94.9 %.

Several previous studies have evaluated other rapid whole blood test kits for *H. pylori* antibody detection, reporting sensitivities and specificities of 80.3–89.5 % and 78.0–93.5 %, and PPVs and NPVs of 83–92.9 % and 57.4–93.5 %, respectively [13, 17–19].

Herein, seventeen patients (15.7 %), who had a negative ^13^C-UBT result were found positive for *H. pylori* antibody by the gabControl^®^*H. pylori* testing. Of these, 16 (94.1 %) individuals had a positive *H. pylori* anamnesis with one or two completed eradication therapies most likely responsible for the relatively low specificity of 76.7 % observed for the gabControl^®^*H. pylori* assay as well as the substantial agreement of 0.62 (95 % CI 0.47–0.76; P < 0.001) between the two methodologies investigated here. Patients with previous eradication therapy might recently have overcome their infection and the ^13^C-UBT, as an indicator of current active infection, might be negative [20, 21]. Furthermore, it is known that it may take more than 1 year for *H. pylori* antibody to disappear after successful eradication [22]. Moreover, post-treatment circulating *H. pylori* antibodies are considered to remain positive for a significant period or perhaps indefinitely in some patients [3, 4, 20].

The intake of proton pump inhibitors (PPIs) may be another possible explanation of discrepant test results between the ^13^C-UBT and the gabControl^®^*H. pylori* assay. While PPI intake at least 2 weeks before the ^13^C-UBT was an exclusion criterion of this study, the authors cannot guarantee, that all individuals strictly followed this instruction. Individuals with PPI intake within 2 weeks before the ^13^C-UBT may have false-negative breath test results, whereas *H. pylori* antibodies are serologically detectable [23–25].

Previous studies, which were performed in various populations with different geographical and socio-economic status, demonstrated, that the prevalence of antibodies against *H. pylori* increases with age [26–28]. A cross-sectional population study in Germany comprising 1797 individuals showed a *H. pylori* antibody prevalence of 48 % [29]. These seroprevalence data are in agreement with our study that found IgG antibodies against *H. pylori* in 49/108 (45.4 %) individuals.

In clinical practice, it should be considered, that a positive IgG serology does not necessarily indicate an ongoing infection [30]. As a consequence the ^13^C-UBT may not be replaced through rapid whole-blood IgG antibody screening tests for confirmation of the successful *H. pylori* eradication after antibiotic treatment.

Using finger-stick blood samples the gabControl^®^*H. pylori* assay is rapid and easy to perform as centrifugation is no necessary and results are available within 10 min. However, low antibody titers resulting in faintest signal intensities may lead to false negative read-outs as interpretation becomes highly subjective and should therefore be based on the four eyes principle.

The major limitation of this study is the lack of invasive biopsy-based methods (e.g. histology, bacterial culture and real-time PCR) for *H. pylori* detection.

In conclusion, the gabControl^®^*H. pylori* immunoassay is a rapid and easy to use first-line screening tool for *H. pylori* IgG antibody detection in daily clinical practice. However, it should not be used for confirmation of the successful *H. pylori* eradication after antibiotic treatment.

## Abbreviations

H: Helicobacter
MALT: Mucosa-associated lymphoid tissue
^13^C-UBT: ^13^C-urea breath test
CO_2_: Carbon dioxide
NH_3_: Ammonia
DOB: Delta over baseline
κ: Kappa
PPV: Positive predictive value
NPV: Negative predictive value
PPI: proton pump inhibitor

## References

[1] Eusebi LH, Zagari RM, Bazzoli F (2014). Epidemiology of *Helicobacter pylori* infection. Helicobacter..

[2] Mentis A, Lehours P, Mégraud F (2015). Epidemiology and diagnosis of *Helicobacter pylori* infection. Helicobacter..

[3] Cutler AF, Prasad VM (1996). Long-term follow-up of *Helicobacter pylori* serology after successful eradication. Am J Gastroenterol.

[4] Sharma TK, Young EL, Miller S, Cutler AF (1997). Evaluation of a rapid, new method for detecting serum IgG antibodies to *Helicobacter pylori*. Clin Chem.

[5] Patel SK, Pratap CB, Jain AK, Gulati AK, Nath G (2014). Diagnosis of *Helicobacter pylori*: what should be the gold standard?. World J Gastroenterol.

[6] Parente F, Bianchi Porro G (2001). The (13)C-urea breath test for non-invasive diagnosis of *Helicobacter pylori* infection: which procedure and which measuring equipment?. Eur J Gastroenterol Hepatol.

[7] Savarino V, Vigneri S, Celle G (1999). The 13C urea breath test in the diagnosis of *Helicobacter pylori* infection. Gut.

[8] Lopes AI, Vale FF, Oleastro M (2014). *Helicobacter pylori* infection—recent developments in diagnosis. World J Gastroenterol.

[9] Epple HJ, Kirstein FW, Bojarski C, Frege J, Fromm M, Riecken EO, Schulzke JD (1997). 13C-urea breath test in *Helicobacter pylori* diagnosis and eradication. Correlation to histology, origin of “false” results, and influence of food intake. Scand J Gastroenterol.

[10] Kawakami E, Machado RS, Reber M, Patrício FR (2002). 13 C-urea breath test with infrared spectroscopy for diagnosing *Helicobacter pylori* infection in children and adolescents. J Pediatr Gastroenterol Nutr.

[11] Blanchard TG, Nedrud JG, Czinn SJ (1999). Local and systematic antibody responses in humans with *Helicobacter pylori* infection. Can J Gastroenterol..

[12] Rathbone BJ, Wyatt JI, Worsley BW, Shires SE, Trejdosiewicz LK, Heatley RV, Losowsky MS (1986). Systemic and local antibody responses to gastric *Campylobacter pyloridis* in non-ulcer dyspepsia. Gut.

[13] Hackelsberger A, Schultze V, Peitz U, Günther T, Nilius M, Diete U, Schumacher M, Roessner A, Malfertheiner P (1998). Performance of a rapid whole blood test for *Helicobacter pylori* in primary care: a German multicenter study. Helicobacter..

[14] Chen TS, Chang FY, Lee SD (2002). No difference of accuracy between capillary and venous blood in rapid whole blood test for diagnosis of *Helicobacter pylori* infection. Dig Dis Sci.

[15] Burucoa C, Delchier JC, Courillon-Mallet A, de Korwin JD, Mégraud F, Zerbib F, Raymond J, Fauchère JL (2013). Comparative evaluation of 29 commercial *Helicobacter pylori* serological kits. Helicobacter..

[16] Viera AJ, Garrett JM (2005). Understanding interobserver agreement: the kappa statistic. Fam Med.

[17] Jones R, Phillips I, Felix G, Tait C (1997). An evaluation of near-patient testing for *Helicobacter pylori* in general practice. Aliment Pharmacol Ther.

[18] Harrison JR, Bevan J, Furth EE, Metz DC (1998). AccuStat whole blood fingerstick test for *Helicobacter pylori* infection: a reliable screening method. J Clin Gastroenterol.

[19] Laine L, Knigge K, Faigel D, Margaret N, Marquis SP, Vartan G, Fennerty MB (1999). Fingerstick *Helicobacter pylori* antibody test: better than laboratory serological testing?. Am J Gastroenterol.

[20] Newell DG, Hawtin PR, Stacey AR, MacDougall MH, Ruddle AC (1991). Estimation of prevalence of *Helicobacter pylori* infection in an asymptomatic elderly population comparing [14C] urea breath test and serology. J Clin Pathol.

[21] Domínguez-Muñoz JE, Leodolter A, Sauerbruch T, Malfertheiner P (1997). A citric acid solution is an optimal test drink in the 13C-urea breath test for the diagnosis of *Helicobacter pylori* infection. Gut.

[22] Kim SG, Jung HK, Lee HL, Jang JY, Lee H, Kim CG, Shin WG, Shin ES, Lee YC, Korean College of Helicobacter and Upper Gastrointestinal Research (2014). Guidelines for the diagnosis and treatment of *Helicobacter pylori* infection in Korea, 2013 revised edition. J Gastroenterol Hepatol.

[23] Laine L, Estrada R, Trujillo M, Knigge K, Fennerty MB (1998). Effect of proton-pump inhibitor therapy on diagnostic testing for *Helicobacter pylori*. Ann Intern Med.

[24] Graham DY, Opekun AR, Hammoud F, Yamaoka Y, Reddy R, Osato MS, El-Zimaity HM (2003). Studies regarding the mechanism of false negative urea breath tests with proton pump inhibitors. Am J Gastroenterol.

[25] Mana F, Van Laer W, Bossuyt A, Urbain D (2005). The early effect of proton pump inhibitor therapy on the accuracy of the 13C-urea breath test. Dig Liver Dis..

[26] Jones DM, Eldridge J, Fox AJ, Sethi P, Whorwell PJ (1986). Antibody to the gastric campylobacter-like organism (“*Campylobacter pyloridis*”)—clinical correlations and distribution in the normal population. J Med Microbiol.

[27] Mégraud F, Brassens-Rabbé MP, Denis F, Belbouri A, Hoa DQ (1989). Seroepidemiology of *Campylobacter pylori* infection in various populations. J Clin Microbiol.

[28] Veldhuyzen van Zanten SJ, Pollak PT, Best LM, Bezanson GS, Marrie T (1994). Increasing prevalence of Helicobacter pylori infection with age: continuous risk of infection in adults rather than cohort effect. J Infect Dis.

[29] Michel A, Pawlita M, Boeing H, Gissmann L, Waterboer T (2014). *Helicobacter pylori* antibody patterns in Germany: a cross-sectional population study. Gut Pathog..

[30] Zagari RM, Romano M, Ojetti V, Stockbrugger R, Gullini S, Annibale B, Farinati F, Ierardi E, Maconi G, Rugge M, Calabrese C, Di Mario F, Luzza F, Pretolani S, Savio A, Gasbarrini G, Caselli M (2015). Guidelines for the management of *Helicobacter pylori* infection in Italy: The III Working Group Consensus Report 2015. Dig Liver Dis..

